# Supplementary material for: HLA-DRB1*15:01 drives sex- and age-dependent microglial activation and neuroimmune signaling
Source: bioRxiv. 2026 Mar 14:2026.03.11.711132. Preprint. [Version 1] doi: 10.64898/2026.03.11.711132 (PMC13061024; doi:10.64898/2026.03.11.711132)
Supplement: Supplement 1 [file media-1.pdf]

Supplementary Figure 1

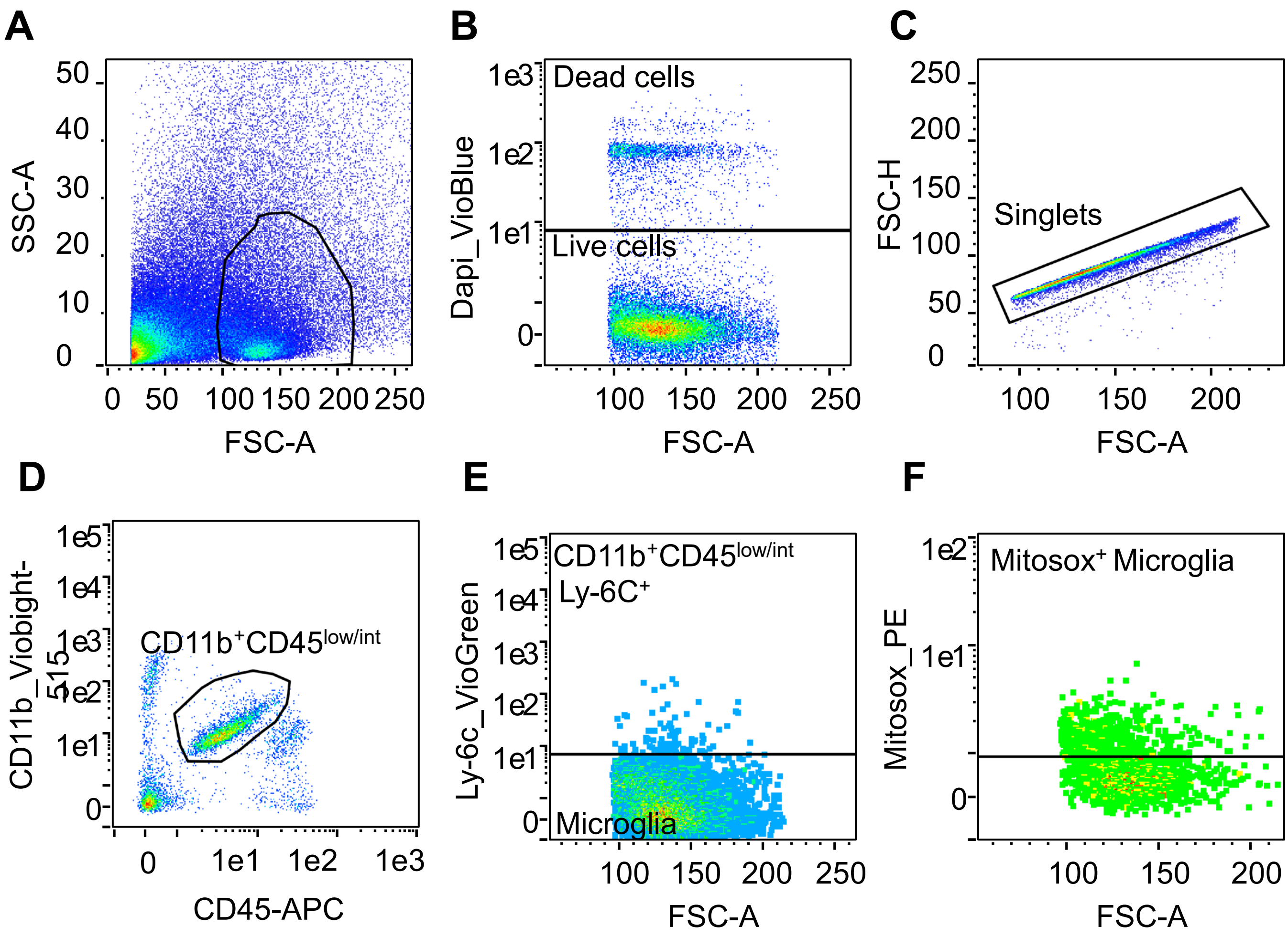

Supplementary Figure 1. Gating strategy for identification of MitoSOX^{+} microglia.
(A) Single-cell suspensions from adult mouse brain were first gated on FSC-A vs SSC-A to exclude debris. (B) Dead cells were excluded using DAPI staining (C) followed by FSC-A/FSC-H gating to select singlets. (D) Microglia were identified as CD11b^{+} CD45$^{low/int}$ cells. (E) Peripheral monocytes were further excluded based on Ly6C expression.(F) Mitochondrial superoxide production was assessed using MitoSOX-PE within the gated microglia population.

Supplementary Figure 2

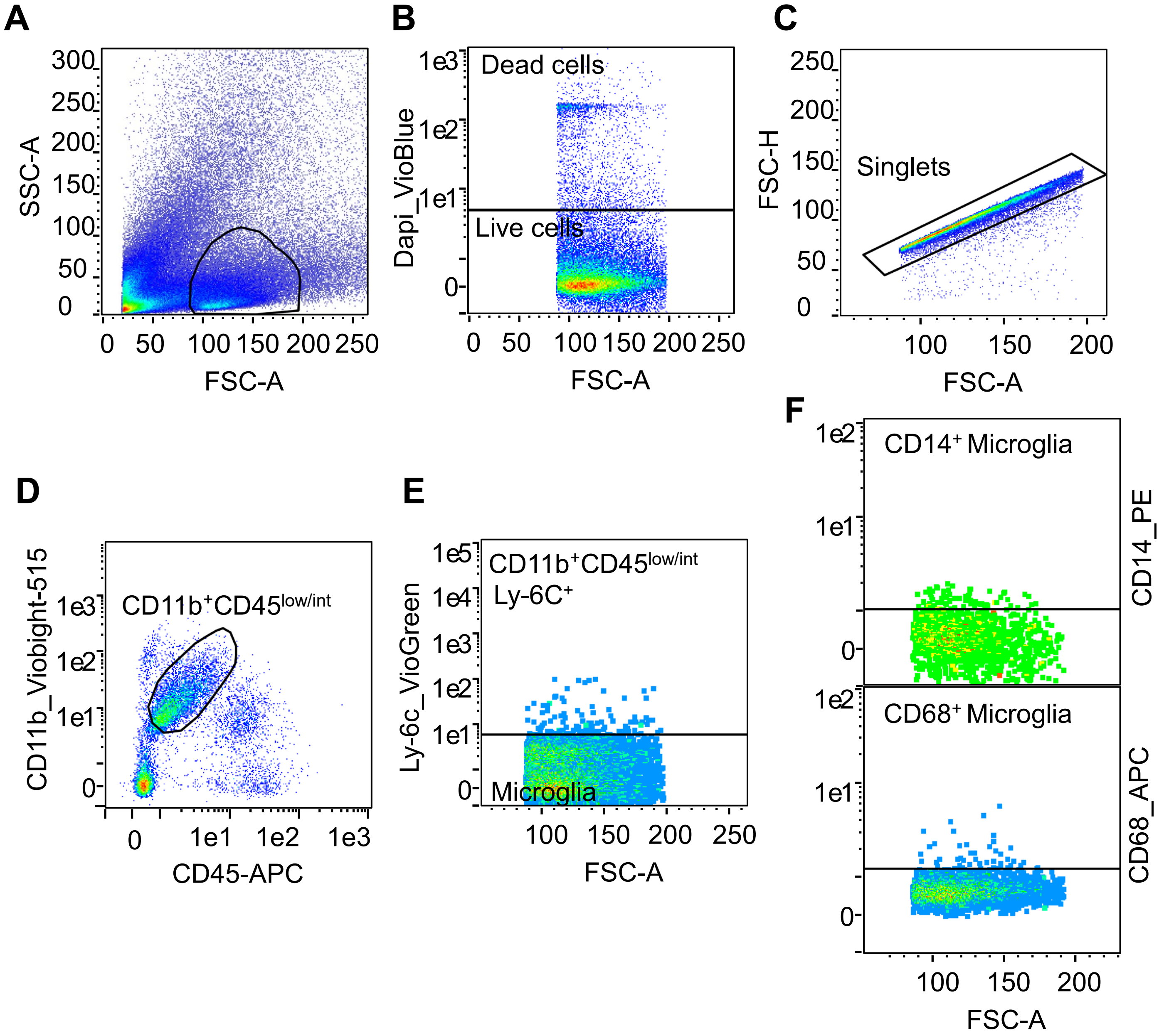

Supplementary Figure 2. Microglia gating and activation analysis by flow cytometry. Brain single-cell suspensions were gated to (A) exclude debris, (B) dead cells (DAPI^{+}, and (C) doublets. (D) Microglia were defined as CD11b^{+} CD45$^{low/int}$ cells, (E) excluding Ly6C^{+} events. Activation was assessed by (F) CD14 and CD68 expression.

Supplementary Figure 3

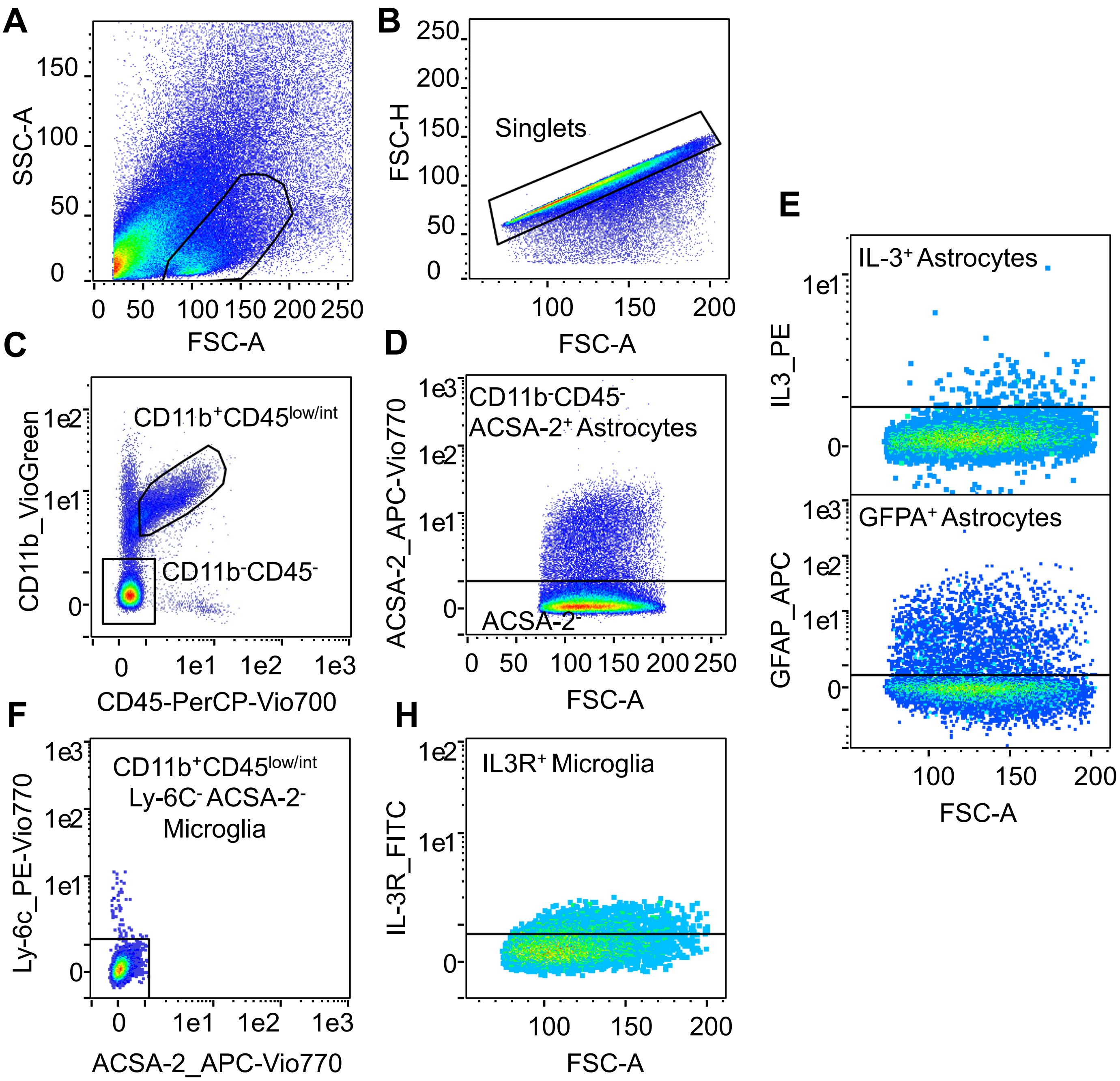

Supplementary Figure 3. Gating strategy for IL-3/IL-3R analysis in astrocytes and microglia. Single-cell suspensions from adult mouse brain were analyzed after fixation. Cells were first gated on (A) FSC-A versus SSC-A to exclude debris, (B) followed by FSC-A/FSC-H gating to select singlets. (C) From singlets, CD11b and CD45 expression were used to separate myeloid($CD11b^{+}CD45^{low/int}$) and non-myeloid populations ($CD11b^{-}CD45^{-}$). (D) The $CD11b^{-}$ $CD45^{-}$ population was used to identify astrocytes based on ACSA-2 expression, and (E) IL-3 and GFAP expression were assessed within the ACSA-2^{+} astrocyte population (D). In parallel, the CD11b^{+} CD45^low/int population (C) was further analyzed by (F) Ly-6C and ACSA-2 expression to define microglia as CD11b^{+} CD45^low/int Ly-6C^{-} ACSA-2^{-} cells. (H) IL-3 receptor (IL-3R) expression was subsequently assessed within the gated microglia population.

Supplementary Figure 4

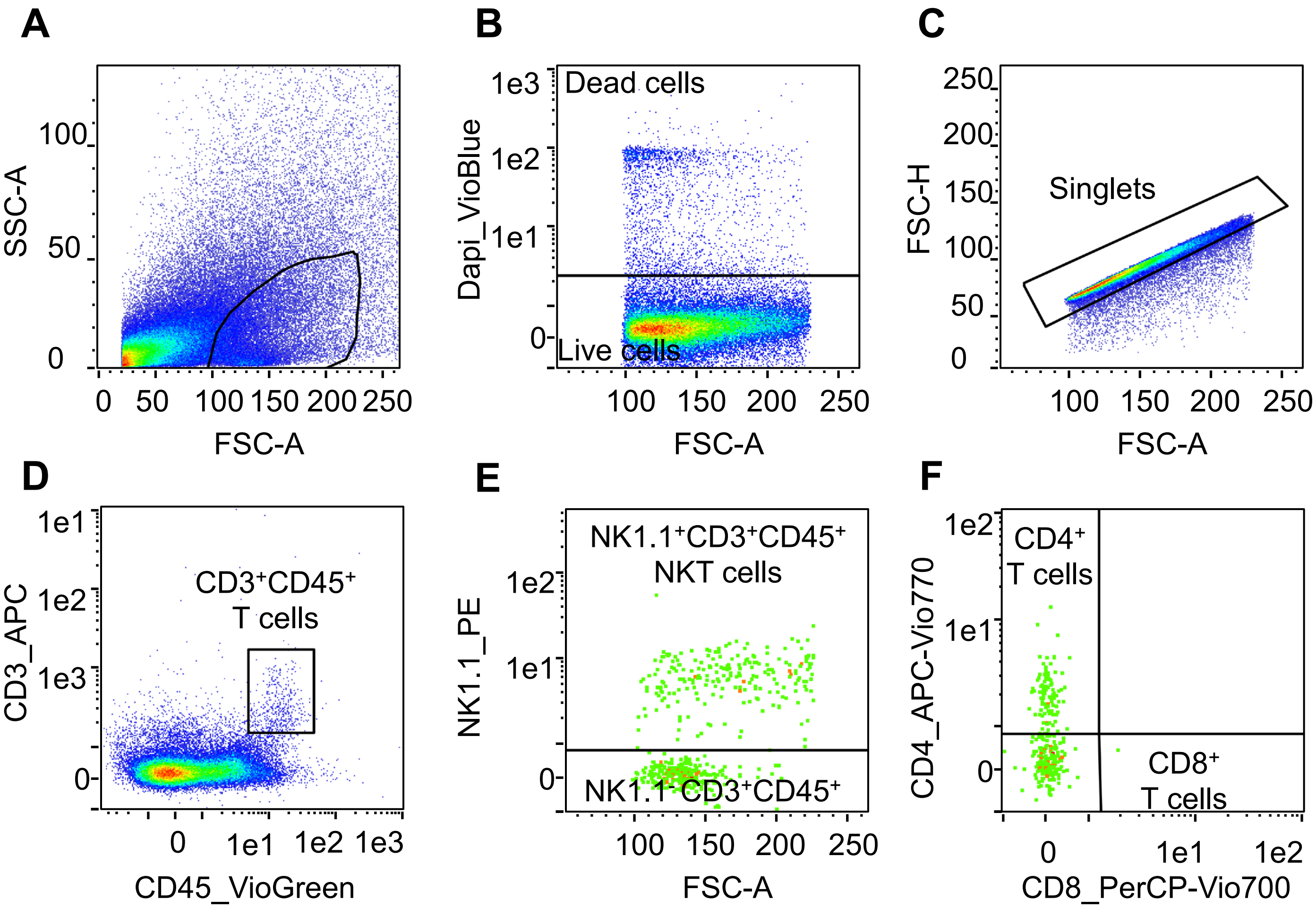

Supplementary Figure 4. Flow cytometric gating strategy for identification of T cell and NKT cell populations in brain and meninges. Single-cell suspensions from mouse brain and meninges were analyzed using the same gating strategy; representative plots shown are from brain. (A) Cells were first gated on FSC-A versus SSC-A to exclude debris. (B) Dead cells were excluded using a viability dye to identify live cells, (C) followed by FSC-A/FSC-H gating to select singlets. (D) From live singlets, CD45 and CD3 expression was used to define T cells ($CD3^+$ $CD45^+$). (E) Within the $CD3^+$ $CD45^+$ population, NKT cells were identified based on NK1.1 expression ($NK1.1^+$ $CD3^+$ $CD45^+$), while $NK1.1^-$ $CD3^+$ $CD45^+$ cells were classified as (F) $CD4^+$ and $CD8^+$ T cell subsets

Supplementary Figure 5

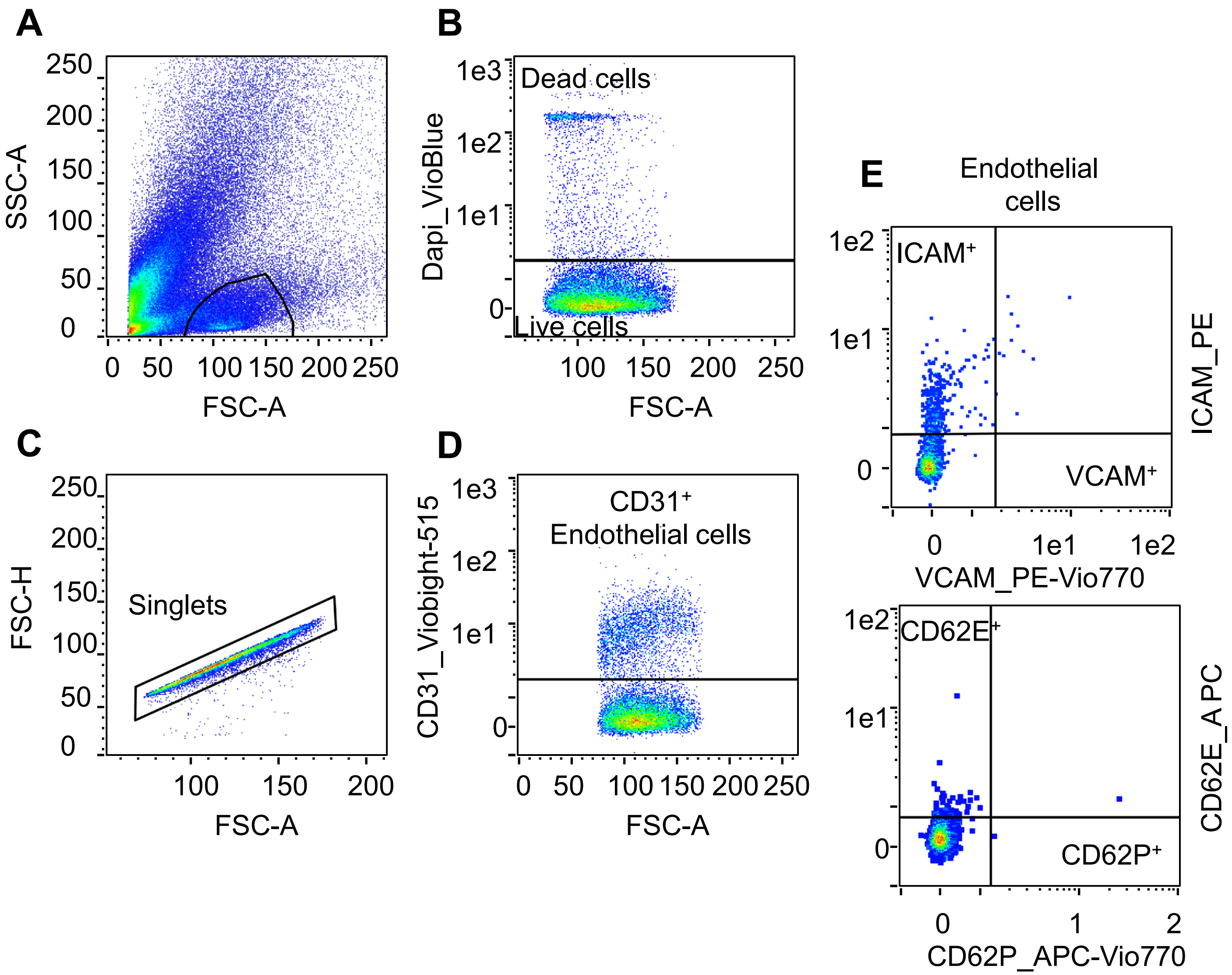

Supplementary Figure 5. Flow cytometric gating strategy for identification of activated endothelial cells in the brain. (A) Single-cell suspensions from mouse brain were first gated on FSC-A versus SSC-A to exclude debris. (B) Dead cells were excluded using a viability dye to identify live cells. (C) FSC-A versus FSC-H gating was used to select singlets. (D) From live singlets, endothelial cells were identified based on CD31 expression. (E) Within the $CD31^+$ population, activated endothelial cells were identified by expression of ICAM-1 ($ICAM\text{-}1^+$ $CD31^+$), VCAM-1 ($VCAM\text{-}1^+$ $CD31^+$), E-selectin ($CD62E^+$ $CD31^+$), and P-selectin ($CD62P^+$ $CD31^+$).
